# Hidden Kiss: A Rare Case of Spontaneous Suprachoroidal Hemorrhage Masquerading as Vitreous Hemorrhage Causing Secondary Angle-Closure Glaucoma

**DOI:** 10.7759/cureus.42817

**Published:** 2023-08-01

**Authors:** Jonathan K. Y. Ng, Antonia Peilober-Richardson, Jae Yee Ku, Kirti Jasani, David Haider

**Affiliations:** 1 Ophthalmology, Manchester Royal Eye Hospital, Manchester University NHS Foundation Trust, Manchester, GBR; 2 Ophthalmology, Royal Bolton Hospital, Bolton NHS Foundation Trust, Bolton, GBR; 3 Eye and Vision Science, Institute of Life Course and Medical Sciences, Faculty of Health & Life Sciences, University of Liverpool, Liverpool, GBR

**Keywords:** vitreous hemorrhage, angle closure glaucoma, secondary glaucoma, spontaneous suprachoroidal hemorrhage, suprachoroidal hemorrhage

## Abstract

Suprachoroidal hemorrhage (SCH) is an uncommon sight-threatening pathology, most often encountered intraoperatively. However, spontaneous presentation of SCH is even rarer. We report the case of a 69-year-old diabetic patient with spontaneous SCH (SSCH) in her left eye masquerading as a vitreous hemorrhage. She developed treatment-resistant secondary angle-closure glaucoma. She was referred to the vitreoretinal team for intraocular exploration to identify the source of the hemorrhage. Pars plana vitrectomy identified extensive SCH intraoperatively. As far as the authors are aware, this is the first case in which the patient had such severe SSCH that the characteristic kissing choroidal sign was not visualized on repeated examinations and multimodal imaging. All initial evidence pointed towards a diagnosis of vitreous hemorrhage. This case demonstrates that if a patient has angle-closure glaucoma and persistently raised intra-ocular pressure that is treatment-resistant, then SCH is an important differential diagnosis to consider. Clinicians need to be aware of the risk factors of SCH, and early recognition with a timely intervention of SCH is important to optimize visual outcomes.

## Introduction

Suprachoroidal hemorrhage (SCH) is an uncommon sight-threatening pathology, most often encountered intraoperatively during incisional intraocular surgery [1,2], and spontaneous manifestation is even rarer [3]. Systemic risk factors include advanced age, hypertension, ischemic heart disease, diabetes mellitus, anticoagulant therapy, chronic kidney disease, and blood dyscrasias [3]. Ocular risk factors include age-related macular degeneration [4], glaucoma [5,6], and high myopia [7]. We report the case of a patient with spontaneous SCH (SSCH), masquerading as vitreous hemorrhage, who developed treatment-resistant secondary angle-closure glaucoma, eventually requiring intraocular exploration as a last resort. The extent of her SSCH was so severe that it masked the classic kissing choroidal sign on B-scan, making this a unique presentation of an already rare event.

## Case presentation

A 69-year-old woman presented with reduced vision in her left eye over five days. Her best corrected visual acuity (BCVA) was 0.20 logMAR OD (Oculus Dexter) and hand movements OS (Oculus Sinister). Her anterior segment examination was unremarkable with normal intraocular pressure (IOP) in both eyes. Fundoscopic examination of her right eye showed moderate non-proliferative diabetic retinopathy while an examination of her left eye showed a dense vitreous hemorrhage. A B-scan of her left eye showed a flat retina. She was presumed to have a vitreous hemorrhage in her left eye secondary to proliferative diabetic retinopathy. She was discharged home with a plan to observe for spontaneous resolution of the vitreous hemorrhage and a follow-up appointment in the medical retina clinic. Her past medical history included asthma, type 2 diabetes mellitus for over 10 years, hypertension, ischemic heart disease, for which she took clopidogrel, and early chronic kidney disease (CKD). She was pseudophakic in both eyes and her last documented BCVA was 0.20 logMAR OU (Oculus Uterque) after routine cataract surgeries. She had no other previous ocular history.

**Figure 1:**
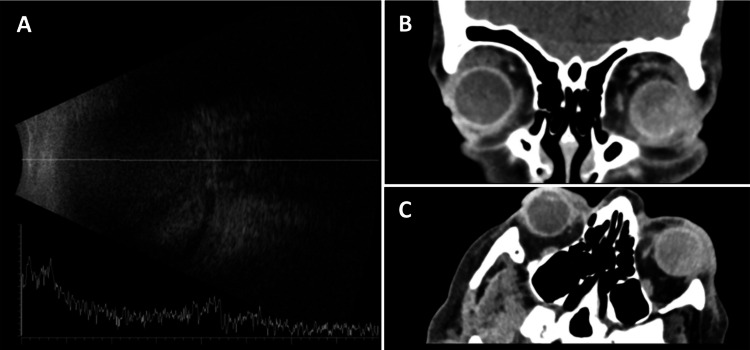
B-scan ultrasonography of the left eye and computerized tomography scans. B-scan ultrasonography of the left eye showed a flat retina with dense hyperechogeneity in the vitreous cavity and an absence of the kissing choroidal sign (A). Computerized tomography (CT) scans (B, coronal plane; C, transverse plane) showed hyperdensity in the posterior segment of the patient's left eye.

She represented with left eye pain three days later. The BCVA in her right eye remained at 0.20 logMAR but the visual acuity (VA) in her left eye had deteriorated to perception of light (PL). Her right eye examination was unchanged. Examination of her left eye showed corneal edema with a shallow anterior chamber, but there was no iridocorneal touch. A gonioscopy examination of her right eye showed open angles. A gonioscopy examination of her left eye was limited by the corneal edema but showed narrowed angles with no obvious peripheral anterior synechiae. Fundoscopic examination of her left eye remained obscured due to the presence of dense hemorrhage in the posterior segment. A repeat B-scan of her left eye showed a flat retina with dense hyperechogenicity within the vitreous cavity (Figure 1A). Intra-ocular pressure was within normal limits in her right eye but raised in her left eye (56 mmHg). She was given maximal topical anti-glaucoma therapy (latanoprost 0.005%, dorzolamide 2%, iopidine 1%) in her left eye. Beta-blockers were withheld due to her background history of asthma. She was also given intravenous (IV) acetazolamide 500 mg with guidance from the medical registrar in view of her CKD. These measures brought her left eye IOP down to 36 mmHg.

**Figure 2:**
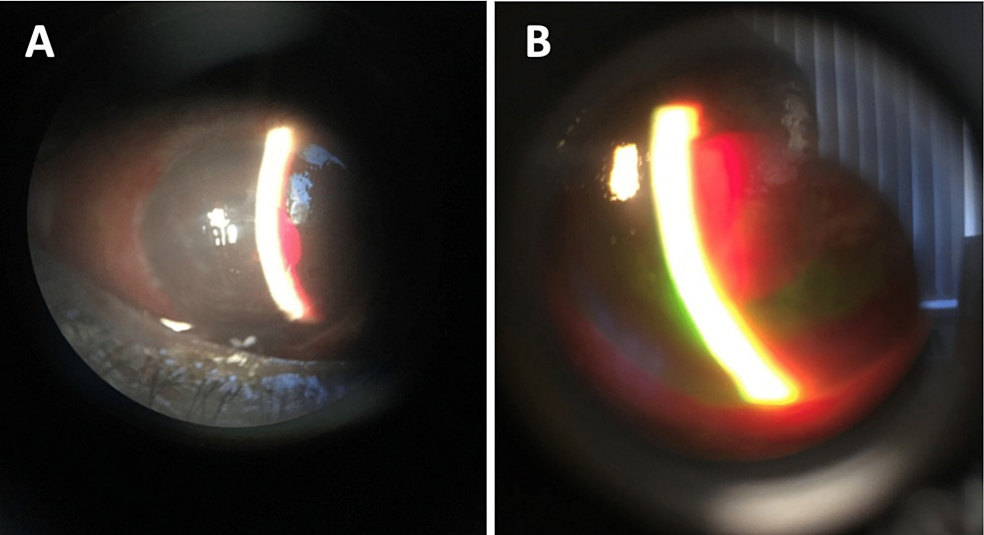
Slit-lamp photographs of the patient’s left eye. A) Corneal edema and bullous keratopathy due to high intraocular pressure with a very shallow anterior chamber whereby there is hardly any space between the slit lamp beam, angled obliquely, and the iris. (B) Dense hemorrhage behind the intraocular lens.

On review the next day, her left eye VA had further deteriorated to no perception of light (NPL) and her left eye IOP had increased to 80 mmHg. Her left eye examination showed increased corneal edema, bullous keratopathy, shallow anterior chamber, and 360° peripheral iridocorneal touch. Her left eye fundal view was still obscured by a dense vitreous hemorrhage (Figures 2A, 2B). A computerized tomography (CT) head was performed and it showed hyperdensity in the posterior segment which supported the initial working diagnosis of vitreous hemorrhage in the patient’s left eye (Figures 1B, 1C). She was given IV mannitol (200 g) under guidance from the medical registrar, which reduced her left eye IOP to 63 mmHg. She also had left eye transscleral cyclophotocoagulation which further lowered her left eye IOP to 50 mmHg.

**Figure 3:**
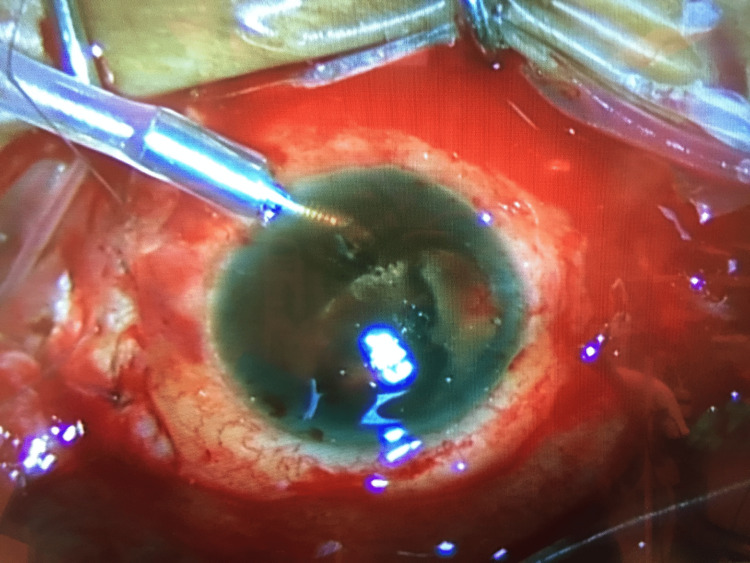
Intraoperative picture of the patient’s left eye. Intraoperative picture of the patient’s left eye during pars plana vitrectomy after clearance of the hemorrhage behind the intraocular lens in the posterior chamber. The retina was seen abutting the intraocular lens due to an extensive suprachoroidal hemorrhage anteriorly at the 2-4 and 6-10 o’clock positions.

The following day, the IOP in her left eye was >80 mmHg and remained persistently high after repeated optimal medical therapy. The patient was referred to the vitreoretinal team for intraocular exploration in an attempt to identify the source of the hemorrhage. At the early stages of the pars plana vitrectomy, there was a mixture of dark blood and clots gushing out from the 25G trocars due to the high IOP. The vitrectomy cutter was inserted into what was thought to be the vitreous cavity and the hemorrhage was cleared and debulked as much as possible. Intra-operatively, the surgeon found that the vitrectomy cutter was in the suprachoroidal cavity clearing the SCH rather than the vitreous cavity. When the extensive nature of the SCH became apparent, the surgery was abandoned due to the poor visual prognosis which cannot justify further interventions (Figure 3).

The patient’s visual outcome remained poor (NPL) in her left eye in all her follow-up appointments. She had chronically raised IOP despite maximally tolerated topical treatment. Eventually, the patient chose to have an evisceration of her left eye three months after her presentation due to intractable pain.

## Discussion

SSCH is an uncommon condition with a recent meta-analysis citing only 32 reported cases over 12 years up to 2016 [8] and a further four cases up to 2022 [9-12]. As far as the authors are aware, this is the first case report whereby the patient had such an extensive SSCH that the typical sign of kissing choroidals was absent. The patient also did not have evidence of SCH on multiple examinations and multimodal imaging. All initial clinical and imaging findings suggested a diagnosis of vitreous hemorrhage until the patient was found to have SSCH during intraoperative exploration.

There are various systemic and ocular conditions associated with an increased risk of SSCH. Some mechanisms have been proposed, and they all appear to play an interconnected role in the pathophysiology of SSCH [8]. Firstly, patients such as the one in our case, who had cardiovascular risk factors including hypertension, diabetes, and atherosclerosis tend to have more fragile vasculature in their choroidal and posterior ciliary vessels. This can lead to a higher likelihood of spontaneous breakage [3,8]. Older age of over 60 years old also predisposes patients to such spontaneous breakage [13]. Secondly, these delicate blood vessels can be damaged by physical forces secondary to changes in IOP, as seen during a Valsalva maneuver or malignant hypertension [14]. When there is a sudden rise in overall blood pressure, this can lead to an elevation in the resistance of blood vessels surrounding the eye, and consequently, an increase in IOP. This pressure is then transferred to the delicate vessels in the choroid, making them susceptible to damage [14,15]. Thirdly, conditions such as blood dyscrasia or being on anticoagulants or antiplatelet medications can cause minor to severe hemorrhaging due to disruption to hemostasis [8], resulting in bleeding through different levels of the retina and choroid. This was the case with our patient who was on clopidogrel.

Given that the pathophysiology of SSCH is multifactorial, patients with multiple risk factors have an increased risk of developing SSCH and our patient had several of these risk factors. Patients’ co-morbidities also posed a challenge when managing raised IOP. In this case, beta-blockers were contraindicated due to her asthma. Acetazolamide and mannitol were used cautiously due to her CKD.

Transscleral cyclophotocoagulation had been previously reported by Shan et al. in treating refractory ocular hypertension in a patient with SSCH. Transscleral cyclophotocoagulation was effective in lowering IOP and controlling pain in the affected eye. Although the patient’s SSCH had partially resolved after two months, the patient’s VA of the affected eye remained at NPL [12]. In our patient, transscleral cyclophotocoagulation only showed transient and modest effects in lowering IOP. This could be due to persistent bleeding from the SSCH, which may have caused a mass effect from the posterior segment displacing the irido-lens diaphragm forward, shallowing the anterior chamber, and closing the angle.

Interestingly, there were two case reports on secondary angle closure from vitreous hemorrhage. One of the cases was from rupture of a retinal arterial macroaneurysm and the other after undergoing intravitreal injection to treat exudative macular degeneration [16,17]. In another case, a patient developed angle-closure glaucoma from a retinal detachment with subretinal and vitreous hemorrhage [18]. All these cases had substantial vitreous hemorrhage which could have caused a mass effect from the posterior segment leading to angle closure from the mechanism described above.

Surgical drainage of the SCH either via sclerotomy or vitrectomy to minimize damage from the suprachoroidal bleed has been reported to preserve some vision for patients [19]. A case series of 51 patients with SCH showed no significant difference in the final VA between those who had surgical intervention versus those who had observation only [20]. In our patient’s case, her VA in the affected eye was already down to NPL and she had persistently raised IOP resistant to medical and laser treatment. Therefore, intraocular exploration was performed to identify the source of the hemorrhage. Her diagnosis before surgical exploration was still uncertain, with the top differential diagnoses being either SSCH or profuse vitreous hemorrhage causing secondary angle closure.

This case also highlights the necessity of careful clinical reasoning and revision of working diagnoses. Patients with SCH generally have poor visual prognosis and there is no consensus for standard management. Early diagnosis with surgery may provide the best chance of restoring some level of vision [1]. Besides preserving sight, the aim of management is to ensure patients’ comfort, and eye removal via evisceration or enucleation may be necessary sometimes. In our patient’s case, the patient had an evisceration to manage her painful blind eye. The fellow eye will be kept under surveillance.

## Conclusions

In conclusion, this case has demonstrated that if a patient has angle closure glaucoma and persistently raised IOP that is treatment-resistant, SCH is an important differential diagnosis to consider. SCH can present without the classic sign of kissing choroidal shown on B-scans. Clinicians need to be aware of the risk factors of SCH. Early recognition and intervention of SCH is important to optimize visual outcome.
